# Strangulated Inguinal Hernia of a Large Portion of Omentum, Cured by an Operation

**Published:** 1846-07

**Authors:** Paul F. Eve

**Affiliations:** Professor of Surgery in the Medical College of Georgia


*Strangulated Inguinal Hernia of a large portion of Omentum, cured by an Operation.* By Paul F. Eve, M. D., Professor of Surgery in the Medical College of Georgia.—On the 17th of last August, I was requested by Drs. Hanson and Jones, of an adjoining county, to see with them a patient labouring under strangulated hernia. Mr. G. S—— is about 44 years, weighs 185 lbs., and is only five feet six inches high: his habits are very good. In 1841, four years ago, while lifting a cotton bale, "he felt something give way in the region of the right groin." On Thursday, the 14th of August, when sowing turnips, he suddenly experienced pain low down in the right side of his abdomen. He took soon after this a dose of salts, which acted freely upon his bowels; but as no relief was thus obtained, Dr. Hanson was sent for, and reached him early on Saturday morning the 16th. Mr. S. was now freely bled, and means employed to reduce a hernia found existing in the right inguinal region. Reduction not being effected, Dr. Jones was sent for, and arrived the evening of the same day. All ordinary means failing to restore the protruding viscus, including tobacco injection, which evacuated the bowels freely, I was sent for at 2, A. M., of the 17th, and saw the patient a few hours afterwards. At half past 8 o'clock, having exhausted taxis, &c., as my two professional friends had already done, the operation was decided upon.

The tumour extended from the external abdominal ring to the bottom of the scrotum on the right side. It was much distended, and the patient complained of great pain at this region. In making the incisions, the *arteria ad cutem abdominis*, was found to require the ligature, and when the sac was opened, a saucer was employed to catch the bloody serum which flowed out. Of this there was more than a half pint, which, together with a portion of omentum, about the size of a man's fist, formed the hernial tumour. There were no adhesions to the sac. The internal abdominal ring was now divided by carrying the edge of Sir Astley Cooper's knife directly upwards, and efforts made to return the protruding portion of omentum. From the induration of the part presented at the internal ring, success did not attend this attempt at reduction. The knife had again to be resorted to, and the ring greatly enlarged by free incisions, and then the omentum only returned by prolonged and forcible manipulations.

After the operation, we concurred in the opinion that our patient, in all probability, would not long survive it. Forty drops of laudanum were prescribed, also absolute diet and quietude. Upon opiates, however, was placed the greatest reliance; and Dr. H., the family physician, kindly consented to remain twenty-four hours with Mr. S.

On the 19th, two days after the operation, I was much gratified to receive a very favourable report from our patient. His sufferings had gradually diminished, his pulse was at 88, and his wound which was now dressed, found to be doing well. We even placed him on another mattrass, while his bed was made up, and his linen changed. He had yet had no evacuation from the bowels, but had passed some flatus. An emollient enema was prescribed, should he be troubled in the bowels during the day, which if not moved on the morrow, were then to be stimulated to action by an injection.

The 1st September I heard Mr. S. was improving, and on the 6th of October, he went eleven miles to vote at our State election. The ligature to the small artery was not removed until the 16th of this month, and during November last I met him in our streets attending to his business.

The soft pad of a truss, with rather a weak spring, was recommended to be worn, in this case, for a few months.—*Ibid.*

